# 2′,3,4,4′,5-Penta­meth­oxy­chalcone

**DOI:** 10.1107/S1600536810040845

**Published:** 2010-10-23

**Authors:** Johannes H. van Tonder, Theunis J. Muller, Barend C. B. Bezuidenhoudt

**Affiliations:** a Department of Chemistry, University of the Free State, PO Box 339, Bloemfontein, 9300, South Africa

## Abstract

In the title chalcone [systemetic name 1-(2,4-dimeth­oxy­phen­yl)-3-(3,4,5-trimeth­oxy­phen­yl)prop-2-en-1-one], C_20_H_22_O_6_, the dihedral angle between the plane of the two benzene rings is 7.03 (4)° with all but one of the meth­oxy groups essentially co-planar with these rings [C—C—O—C torsion angles = −76.1 (2), −0.7 (3), 1.8 (3), −6.2 (3), 2.0 (3)°]. An intra­molecular C—H⋯O inter­action occurs. The crystal packing is stabilized by weak inter­molecular C—H⋯O hydrogen bonds.

## Related literature

For standard bond lengths, see: Allen *et al.* (1987 ▶). For related structures, see: Patil *et al.* (2006 ▶); van Tonder *et al.* (2010 ▶); Teh *et al.* (2006 ▶); Rosli *et al.* (2006 ▶). For the synthesis of the title compound, see: Kraus & Roy (2008 ▶). For the biological activity of flavonoids, see: Pietta *et al.* (2003 ▶). For non-linear optical (NLO) properties of chalcones, see: Marais *et al.* (2005) ▶; Uchida *et al.* (1998) ▶; Kitaoka *et al.* (1990 ▶); Goto *et al.* (1991 ▶); Zhang *et al.* (1990 ▶). For applications of NLO crystals, see: Chemla & Zyss (1987 ▶).
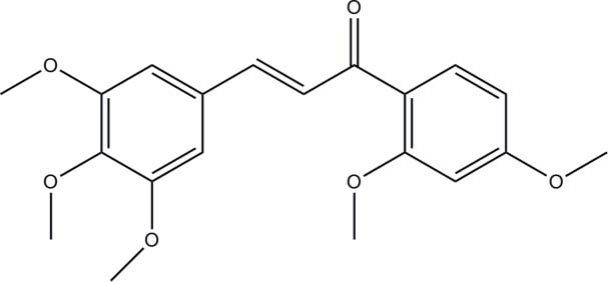


## Experimental

### 

#### Crystal data

C_20_H_22_O_6_
*M* *_r_* = 358.38
Orthorhombic, 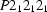

*a* = 7.3041 (2) Å
*b* = 8.0288 (3) Å
*c* = 29.217 (1) Å
*V* = 1713.38 (10) Å^3^
*Z* = 4
Mo *K*α radiation
μ = 0.10 mm^−1^
*T* = 100 K
0.40 × 0.28 × 0.27 mm

#### Data collection

Bruker APEXII CCD area-detector diffractometer
Absorption correction: multi-scan (*SADABS*; Bruker, 2008 ▶) *T*_min_ = 0.960, *T*_max_ = 0.973
30899 measured reflections
2488 independent reflections
2343 reflections with *I* > 2σ(*I*)
*R*_int_ = 0.049

#### Refinement

*R*[*F* ^2^ > 2σ(*F* ^2^)] = 0.032
*wR*(*F* ^2^) = 0.113
*S* = 1.21
2488 reflections
241 parameters
H-atom parameters constrained
Δρ_max_ = 0.47 e Å^−3^
Δρ_min_ = −0.44 e Å^−3^

### 

Data collection: *APEX2* (Bruker, 2008 ▶); cell refinement: *SAINT-Plus* (Bruker, 2008 ▶); data reduction: *SAINT-Plus* and *XPREP* (Bruker, 2008 ▶); program(s) used to solve structure: *SHELXS97* (Sheldrick, 2008 ▶); program(s) used to refine structure: *SHELXL97* (Sheldrick, 2008 ▶); molecular graphics: *DIAMOND* (Brandenberg & Putz, 2005 ▶); software used to prepare material for publication: WingGX (Farrugia, 1999 ▶).

## Supplementary Material

Crystal structure: contains datablocks global, I. DOI: 10.1107/S1600536810040845/zs2069sup1.cif

Structure factors: contains datablocks I. DOI: 10.1107/S1600536810040845/zs2069Isup2.hkl

Additional supplementary materials:  crystallographic information; 3D view; checkCIF report

## Figures and Tables

**Table 1:** Hydrogen-bond geometry (Å, °)

*D*—H⋯*A*	*D*—H	H⋯*A*	*D*⋯*A*	*D*—H⋯*A*
C8—H8⋯O1	0.93	2.13	2.798 (2)	127
C11—H11⋯O3^i^	0.93	2.4	3.306 (2)	164
C17—H17*B*⋯O4^ii^	0.96	2.52	3.455 (2)	165

Symmetry codes: (i) 
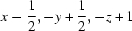
; (ii) 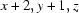.

**Fig. 1.**
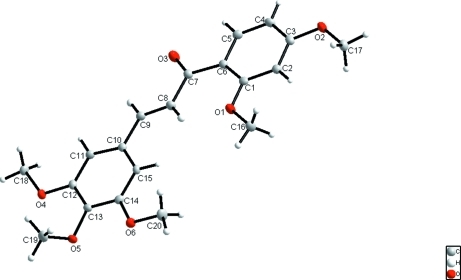
Molecular conformation of the title compound, showing the atom numbering scheme and displacement ellipsoids (50% probability).

## supplementary crystallographic information

### Comment

Flavonoids are a prominent class of secondary plant metabolites known for their wide range of biological activity that include antibacterial, antifungal, anti-tumor and anti-inflammatory properties (Pietta *et al.*, 2003). Chalcones are an important subclass of these compounds and are often utilized as intermediates in the synthesis of a variety of cyclic flavonoids (Marais *et al.*, 2005). Furthermore, many chalcone derivatives are a class of organic compounds with excellent NLO properties (Kitaoka *et al.*, 1990; Goto *et al.*, 1991; Zhang *et al.*, 1990; Uchida *et al.*, 1998; Patil *et al.*, 2006), much better than those observed in inorganic crystals. Nonlinear optical materials capable of generating second harmonic frequency play an important role in the domain of optoelectronics and photonics (Rosli *et al.*, 2006). NLO crystals with high conversion efficiencies for second harmonic generation (SHG) and which are transparent in the visible and ultraviolet ranges are required for numerous device applications (Chemla & Zyss, 1987). Advantages of using organic molecules as NLO materials stem from the fact that they can be designed to optimize the desired NLO properties. At the molecular level, compounds likely to exhibit larger values of molecular hyperpolarizability (*β*), must have polarizable electrons (e.g. π electrons) spread over a large distance. We report here the synthesis and structure of a new chalcone, the title compound C_20_H_22_O_6_, (I).

Crystals of (I) exhibit second-order nonlinear optical properties. Bond distances in (I) have normal values (Allen *et al.*, 1987) and these and bond angles are comparable to those in related structures (van Tonder *et al.*, 2010; Teh *et al.*, 2006; Patil *et al.*, 2006; Rosli *et al.*, 2006). The least-squares plane through the enone group (C7, C8, C9 and O3) exhibit dihedral angles of 5.25 (5)° and 3.32 (6)° with the C1—C6 and C10—C15 benzene rings, respectively. The dihedral angle between the two benzene rings is 7.03 (4)°. The methoxy group attached at C13 is twisted away from the C10—C15 benzene ring plane, with a C19—O5—C13—C12 torsion angle of -76.1 (2)°. The methoxy groups at C1, C3, C12 and C14 are almost coplanar with the C1—C6 and C10—C15 benzene rings with C16—O1—C1—C2, C17—O2—C3—C2, C18—O4—C12—C11 and C20—O6—C14—C15 torsion angles of -0.7 (3), 1.8 (3), -6.2 (3) and 2.0 (3)°, respectively. The C8—C9 bond [1.332 (3) Å] is significantly shorter than the C6—C7, C7—C8 and C9—C10 bonds [1.498 (3), 1.483 (3) and 1.473 (2) respectively]. The C8—C9 bond length indicates a double bond rather than single bonds as in the other bonds. This corresponds well with literature values (Van Tonder *et al.* 2010). Intramolecular C5—H5···O3, C8—H8···O1, C9—H9···O3 and C19—H19C···O4 interactions are found in the molecular structure of (I), while the molecules form chains through weak intermolecular C11—H11···O3^i^ and C17—H17B···O4^ii^ hydrogen bonds (Table 1).

### Experimental

Freshly ground KOH (15.1 g; 270 mmol; 32 eq.) was added to a cold (ice bath) stirred solution of 2',4'-dimethoxyacetophenone (1.52 g; 8.4 mmol) and 3,4,5-trimethoxybenzaldehyde (1.95 g; 10.0 mmol; 1.2 eq.) in ethanol (60 ml). The temperature of the reaction mixture was allowed to rise to room temperature and stirring contined until completion of the reaction (TLC). Extraction of the water phase with ethyl acetate (3 *x* 100 ml) followed by neutralization with aqueous NaHCO_3_ (litmus) and washing with water gave the crude chalcone after drying of the solution (Na_2_SO_4_) and evaporation of the solvent *in vacuo* at *ca.* 40° C. Crystallization from ethanol afforded the desired chalcone (2.15 g; 71.2%) as yellow needles. *R_f_* 0.17 (H:A; 8:2); m.p. 85.1° C; ^1^H NMR (600 MHz, CDCl_3_) δ 7.70 (1*H*, d, *J* = 8.61 Hz, H-6'), 7.54 (1*H*, d, *J* = 15.72 Hz, H-β), 7.35 (1*H*, d, *J* = 15.72 Hz, H-α), 6.79 (2*H*, s, H-2,6), 6.53 (1*H*, dd, *J* = 2.27, 8.61 Hz, H-5'), 6.47 (1*H*, d, *J* = 2.27 Hz, H-3'), 3.86 (9*H*, s, –OCH_3_), 3.85 (3*H*, s, –OCH_3_), 3.83 (3*H*, s, –OCH_3_); ^13^C NMR (151 MHz, CDCl_3_) δ 190.47 (CO), 164.13, 160.31, 153.39, 142.23 (C-β), 139.97, 132.72 (C-6'), 131.01, 126.68 (C-α), 122.21, 105.49 (C-2,6), 105.23 (C-5'), 98.70 (C-3'), 60.96 (–OCH_3_), 56.15 (–OCH_3_), 55.75 (–OCH_3_), 55.54 (–OCH_3_).

### Refinement

The H atoms were placed in geometrically idealized positions and constrained to ride on their parent atoms with with C—H(aromatic and methine) = 0.93 Å and C–H(methyl) = 0.96 ° and U_iso_(H) = 1.2U_eq_(C)(aromatic and methine) or 1.5U<i/>_eq_(C)(methyl). The absolute structure parameter is meaningless and has been removed from the CIF file. Friedel pairs were therefore averaged in the final cycles of the refinement.

### Crystal data

**Table:**

C_20_H_22_O_6_	*D*_x_ = 1.389 Mg m^−^^3^
*M**_r_* = 358.38	Melting point: 358.1 K
Orthorhombic, *P*2_1_2_1_2_1_	Mo *K*α radiation, λ = 0.71073 Å
Hall symbol: P 2ac 2ab	Cell parameters from 9978 reflections
*a* = 7.3041 (2) Å	θ = 2.8–28.2°
*b* = 8.0288 (3) Å	µ = 0.10 mm^−^^1^
*c* = 29.217 (1) Å	*T* = 100 K
*V* = 1713.38 (10) Å^3^	Cuboid, colourless
*Z* = 4	0.40 × 0.28 × 0.27 mm
*F*(000) = 760	

### Data collection

**Table:**

Bruker APEXII CCD area-detector diffractometer	2343 reflections with *I* > 2σ(*I*)
graphite	*R*_int_ = 0.049
φ and ω scans	θ_max_ = 28.4°, θ_min_ = 1.4°
Absorption correction: multi-scan (*SADABS*; Bruker, 2008)	*h* = −9→9
*T*_min_ = 0.960, *T*_max_ = 0.973	*k* = −10→10
30899 measured reflections	*l* = −39→39
2488 independent reflections	

### Refinement

**Table:**

Refinement on *F*^2^	Primary atom site location: structure-invariant direct methods
Least-squares matrix: full	Secondary atom site location: difference Fourier map
*R*[*F*^2^ > 2σ(*F*^2^)] = 0.032	Hydrogen site location: inferred from neighbouring sites
*wR*(*F*^2^) = 0.113	H-atom parameters constrained
*S* = 1.21	*w* = 1/[σ^2^(*F*_o_^2^) + (0.0721*P*)^2^ + 0.1972*P*] where *P* = (*F*_o_^2^ + 2*F*_c_^2^)/3
2488 reflections	(Δ/σ)_max_ = 0.001
241 parameters	Δρ_max_ = 0.47 e Å^−^^3^
0 restraints	Δρ_min_ = −0.44 e Å^−^^3^

### Special details

**Table:**

Geometry. All s.u.'s (except the s.u. in the dihedral angle between two l.s. planes) are estimated using the full covariance matrix. The cell s.u.'s are taken into account individually in the estimation of s.u.'s in distances, angles and torsion angles; correlations between s.u.'s in cell parameters are only used when they are defined by crystal symmetry. An approximate (isotropic) treatment of cell s.u.'s is used for estimating s.u.'s involving l.s. planes.
Refinement. Refinement of *F*^2^ against ALL reflections. The weighted *R*-factor *wR* and goodness of fit *S* are based on *F*^2^, conventional *R*-factors *R* are based on *F*, with *F* set to zero for negative *F*^2^. The threshold expression of *F*^2^ > 2σ(*F*^2^) is used only for calculating *R*-factors(gt) *etc*. and is not relevant to the choice of reflections for refinement. *R*-factors based on *F*^2^ are statistically about twice as large as those based on *F*, and *R*- factors based on ALL data will be even larger.

### Fractional atomic coordinates and isotropic or equivalent isotropic displacement parameters (Å^2^)

**Table:**

	*x*	*y*	*z*	*U*_iso_*/*U*_eq_	
C1	0.6645 (3)	0.6440 (2)	0.61028 (6)	0.0137 (4)	
C2	0.8307 (3)	0.7258 (2)	0.61820 (6)	0.0148 (4)	
H2	0.8557	0.7704	0.6469	0.018*	
C3	0.9589 (3)	0.7405 (2)	0.58310 (6)	0.0154 (4)	
C4	0.9214 (3)	0.6751 (2)	0.53980 (6)	0.0165 (4)	
H4	1.0058	0.6862	0.5162	0.02*	
C5	0.7583 (3)	0.5942 (2)	0.53265 (6)	0.0152 (4)	
H5	0.7347	0.5508	0.5037	0.018*	
C6	0.6251 (3)	0.5738 (2)	0.56704 (6)	0.0133 (4)	
C7	0.4581 (3)	0.4776 (2)	0.55344 (6)	0.0145 (4)	
C8	0.3112 (3)	0.4413 (2)	0.58701 (6)	0.0156 (4)	
H8	0.3191	0.4864	0.6163	0.019*	
C9	0.1683 (3)	0.3459 (2)	0.57636 (6)	0.0145 (4)	
H9	0.1619	0.3042	0.5467	0.017*	
C10	0.0196 (3)	0.3017 (2)	0.60816 (6)	0.0128 (3)	
C11	−0.1257 (3)	0.2052 (2)	0.59173 (6)	0.0132 (4)	
H11	−0.1295	0.1746	0.5611	0.016*	
C12	−0.2650 (3)	0.1548 (2)	0.62144 (6)	0.0130 (3)	
C13	−0.2581 (3)	0.1996 (2)	0.66793 (6)	0.0123 (4)	
C14	−0.1169 (3)	0.3024 (2)	0.68353 (6)	0.0127 (3)	
C15	0.0224 (3)	0.3523 (2)	0.65412 (6)	0.0125 (3)	
H15	0.1172	0.419	0.6649	0.015*	
C16	0.5751 (3)	0.7012 (3)	0.68799 (6)	0.0193 (4)	
H16A	0.6818	0.6487	0.7007	0.029*	
H16B	0.4725	0.6832	0.7079	0.029*	
H16C	0.5968	0.8186	0.6849	0.029*	
C17	1.1706 (3)	0.8786 (3)	0.63268 (6)	0.0187 (4)	
H17A	1.0857	0.9647	0.641	0.028*	
H17B	1.2925	0.9233	0.6322	0.028*	
H17C	1.1642	0.7899	0.6547	0.028*	
C18	−0.4316 (3)	0.0238 (3)	0.56113 (6)	0.0166 (4)	
H18A	−0.4463	0.1259	0.5444	0.025*	
H18B	−0.5374	−0.0452	0.5567	0.025*	
H18C	−0.3246	−0.0335	0.5503	0.025*	
C19	−0.5600 (3)	0.2182 (3)	0.69496 (7)	0.0201 (4)	
H19A	−0.5508	0.3306	0.7059	0.03*	
H19B	−0.6478	0.1584	0.7131	0.03*	
H19C	−0.5986	0.2189	0.6636	0.03*	
C20	0.0107 (3)	0.4563 (3)	0.74565 (7)	0.0213 (4)	
H20A	0.1272	0.4011	0.7443	0.032*	
H20B	−0.016	0.486	0.7768	0.032*	
H20C	0.0143	0.555	0.7272	0.032*	
O1	0.5368 (2)	0.63145 (19)	0.64410 (4)	0.0188 (3)	
O2	1.1249 (2)	0.81565 (19)	0.58822 (5)	0.0185 (3)	
O3	0.4459 (2)	0.4251 (2)	0.51410 (5)	0.0239 (4)	
O4	−0.41125 (19)	0.06034 (18)	0.60910 (4)	0.0154 (3)	
O5	−0.38532 (19)	0.13860 (18)	0.69848 (4)	0.0154 (3)	
O6	−0.12802 (19)	0.34736 (18)	0.72892 (4)	0.0160 (3)	

### Atomic displacement parameters (Å^2^)

**Table:**

	*U*^11^	*U*^22^	*U*^33^	*U*^12^	*U*^13^	*U*^23^
C1	0.0133 (8)	0.0133 (8)	0.0145 (8)	0.0019 (7)	0.0011 (7)	0.0027 (7)
C2	0.0145 (9)	0.0153 (8)	0.0148 (8)	−0.0001 (7)	0.0002 (7)	0.0012 (7)
C3	0.0126 (9)	0.0140 (8)	0.0195 (8)	−0.0011 (7)	0.0002 (7)	0.0018 (7)
C4	0.0150 (8)	0.0184 (9)	0.0160 (8)	0.0003 (8)	0.0041 (7)	0.0010 (7)
C5	0.0168 (9)	0.0165 (9)	0.0124 (8)	0.0010 (7)	0.0017 (7)	0.0003 (7)
C6	0.0117 (8)	0.0135 (8)	0.0148 (8)	0.0007 (7)	0.0003 (6)	0.0021 (7)
C7	0.0140 (9)	0.0153 (8)	0.0142 (8)	−0.0010 (7)	0.0008 (7)	0.0010 (7)
C8	0.0140 (8)	0.0188 (9)	0.0140 (8)	−0.0005 (8)	0.0022 (7)	0.0000 (7)
C9	0.0140 (9)	0.0174 (8)	0.0120 (7)	−0.0001 (7)	0.0006 (6)	0.0009 (7)
C10	0.0114 (8)	0.0128 (8)	0.0141 (8)	0.0003 (7)	−0.0008 (6)	0.0008 (6)
C11	0.0139 (9)	0.0150 (8)	0.0107 (7)	−0.0002 (7)	0.0001 (6)	−0.0003 (7)
C12	0.0117 (8)	0.0121 (8)	0.0153 (8)	−0.0002 (7)	−0.0009 (7)	−0.0003 (7)
C13	0.0110 (8)	0.0138 (8)	0.0121 (8)	0.0002 (7)	0.0010 (6)	0.0024 (7)
C14	0.0144 (9)	0.0123 (8)	0.0116 (7)	0.0021 (7)	−0.0005 (6)	−0.0006 (6)
C15	0.0110 (8)	0.0131 (8)	0.0135 (8)	−0.0014 (7)	−0.0016 (6)	−0.0007 (6)
C16	0.0191 (10)	0.0262 (10)	0.0127 (8)	−0.0054 (9)	0.0012 (7)	−0.0029 (7)
C17	0.0146 (9)	0.0215 (10)	0.0199 (9)	−0.0030 (8)	−0.0017 (7)	−0.0021 (7)
C18	0.0167 (9)	0.0198 (9)	0.0134 (8)	−0.0014 (8)	−0.0033 (7)	−0.0025 (7)
C19	0.0127 (9)	0.0282 (10)	0.0193 (9)	0.0004 (8)	0.0024 (7)	−0.0003 (8)
C20	0.0225 (10)	0.0263 (10)	0.0152 (8)	−0.0049 (9)	−0.0007 (7)	−0.0066 (8)
O1	0.0162 (7)	0.0273 (7)	0.0130 (6)	−0.0067 (6)	0.0030 (5)	−0.0038 (5)
O2	0.0135 (7)	0.0235 (7)	0.0187 (6)	−0.0052 (6)	0.0026 (5)	−0.0016 (6)
O3	0.0227 (8)	0.0333 (9)	0.0158 (6)	−0.0109 (7)	0.0020 (5)	−0.0044 (6)
O4	0.0133 (6)	0.0195 (7)	0.0132 (6)	−0.0044 (6)	−0.0010 (5)	−0.0017 (5)
O5	0.0125 (6)	0.0189 (6)	0.0149 (6)	−0.0009 (6)	0.0031 (5)	0.0030 (5)
O6	0.0173 (7)	0.0198 (7)	0.0108 (6)	−0.0028 (6)	0.0004 (5)	−0.0026 (5)

### Geometric parameters (Å, °)

**Table:**

C1—O1	1.362 (2)	C13—C14	1.398 (3)
C1—C2	1.400 (3)	C14—O6	1.377 (2)
C1—C6	1.413 (3)	C14—C15	1.390 (3)
C2—C3	1.394 (3)	C15—H15	0.93
C2—H2	0.93	C16—O1	1.427 (2)
C3—O2	1.363 (2)	C16—H16A	0.96
C3—C4	1.397 (3)	C16—H16B	0.96
C4—C5	1.373 (3)	C16—H16C	0.96
C4—H4	0.93	C17—O2	1.433 (2)
C5—C6	1.408 (2)	C17—H17A	0.96
C5—H5	0.93	C17—H17B	0.96
C6—C7	1.498 (3)	C17—H17C	0.96
C7—O3	1.227 (2)	C18—O4	1.439 (2)
C7—C8	1.483 (3)	C18—H18A	0.96
C8—C9	1.332 (3)	C18—H18B	0.96
C8—H8	0.93	C18—H18C	0.96
C9—C10	1.473 (2)	C19—O5	1.430 (2)
C9—H9	0.93	C19—H19A	0.96
C10—C11	1.398 (3)	C19—H19B	0.96
C10—C15	1.403 (2)	C19—H19C	0.96
C11—C12	1.397 (3)	C20—O6	1.425 (3)
C11—H11	0.93	C20—H20A	0.96
C12—O4	1.359 (2)	C20—H20B	0.96
C12—C13	1.406 (2)	C20—H20C	0.96
C13—O5	1.378 (2)		
			
O1—C1—C2	120.57 (16)	O6—C14—C13	115.16 (16)
O1—C1—C6	118.67 (16)	C15—C14—C13	120.58 (16)
C2—C1—C6	120.75 (17)	C14—C15—C10	119.79 (17)
C3—C2—C1	120.05 (17)	C14—C15—H15	120.1
C3—C2—H2	120	C10—C15—H15	120.1
C1—C2—H2	120	O1—C16—H16A	109.5
O2—C3—C2	123.66 (17)	O1—C16—H16B	109.5
O2—C3—C4	116.11 (17)	H16A—C16—H16B	109.5
C2—C3—C4	120.23 (17)	O1—C16—H16C	109.5
C5—C4—C3	119.04 (17)	H16A—C16—H16C	109.5
C5—C4—H4	120.5	H16B—C16—H16C	109.5
C3—C4—H4	120.5	O2—C17—H17A	109.5
C4—C5—C6	123.08 (17)	O2—C17—H17B	109.5
C4—C5—H5	118.5	H17A—C17—H17B	109.5
C6—C5—H5	118.5	O2—C17—H17C	109.5
C5—C6—C1	116.83 (17)	H17A—C17—H17C	109.5
C5—C6—C7	115.67 (16)	H17B—C17—H17C	109.5
C1—C6—C7	127.49 (17)	O4—C18—H18A	109.5
O3—C7—C8	119.95 (18)	O4—C18—H18B	109.5
O3—C7—C6	119.01 (17)	H18A—C18—H18B	109.5
C8—C7—C6	121.01 (16)	O4—C18—H18C	109.5
C9—C8—C7	121.71 (16)	H18A—C18—H18C	109.5
C9—C8—H8	119.1	H18B—C18—H18C	109.5
C7—C8—H8	119.1	O5—C19—H19A	109.5
C8—C9—C10	124.71 (17)	O5—C19—H19B	109.5
C8—C9—H9	117.6	H19A—C19—H19B	109.5
C10—C9—H9	117.6	O5—C19—H19C	109.5
C11—C10—C15	120.02 (16)	H19A—C19—H19C	109.5
C11—C10—C9	118.46 (16)	H19B—C19—H19C	109.5
C15—C10—C9	121.52 (17)	O6—C20—H20A	109.5
C12—C11—C10	119.98 (16)	O6—C20—H20B	109.5
C12—C11—H11	120	H20A—C20—H20B	109.5
C10—C11—H11	120	O6—C20—H20C	109.5
O4—C12—C11	124.71 (16)	H20A—C20—H20C	109.5
O4—C12—C13	115.28 (16)	H20B—C20—H20C	109.5
C11—C12—C13	120.01 (17)	C1—O1—C16	119.25 (15)
O5—C13—C14	119.75 (16)	C3—O2—C17	117.57 (15)
O5—C13—C12	120.72 (16)	C12—O4—C18	116.94 (14)
C14—C13—C12	119.49 (16)	C13—O5—C19	113.32 (14)
O6—C14—C15	124.26 (17)	C14—O6—C20	116.70 (15)
			
C16—O1—C1—C2	−0.7 (3)	C5—C6—C7—C8	177.43 (16)
C16—O1—C1—C6	179.75 (17)	C1—C6—C7—O3	180.00 (18)
C17—O2—C3—C2	1.8 (3)	C1—C6—C7—C8	−1.8 (3)
C17—O2—C3—C4	−177.72 (17)	O3—C7—C8—C9	3.1 (3)
C18—O4—C12—C13	174.86 (17)	C6—C7—C8—C9	−175.10 (17)
C18—O4—C12—C11	−6.2 (3)	C7—C8—C9—C10	178.64 (17)
C19—O5—C13—C14	106.1 (2)	C8—C9—C10—C11	177.67 (18)
C19—O5—C13—C12	−76.1 (2)	C8—C9—C10—C15	−3.1 (3)
C20—O6—C14—C13	−178.15 (17)	C9—C10—C15—C14	−177.41 (17)
C20—O6—C14—C15	2.0 (3)	C15—C10—C11—C12	−2.0 (3)
C2—C1—C6—C7	177.63 (17)	C11—C10—C15—C14	1.8 (3)
O1—C1—C6—C7	−2.8 (3)	C9—C10—C11—C12	177.18 (16)
O1—C1—C2—C3	−178.83 (16)	C10—C11—C12—C13	−0.7 (3)
C2—C1—C6—C5	−1.5 (3)	C10—C11—C12—O4	−179.58 (17)
O1—C1—C6—C5	178.03 (16)	O4—C12—C13—O5	4.9 (3)
C6—C1—C2—C3	0.7 (3)	O4—C12—C13—C14	−177.38 (16)
C1—C2—C3—O2	−178.85 (16)	C11—C12—C13—C14	3.6 (3)
C1—C2—C3—C4	0.6 (3)	C11—C12—C13—O5	−174.14 (16)
O2—C3—C4—C5	178.45 (16)	O5—C13—C14—O6	−6.0 (2)
C2—C3—C4—C5	−1.0 (3)	C12—C13—C14—C15	−3.9 (3)
C3—C4—C5—C6	0.2 (3)	O5—C13—C14—C15	173.89 (16)
C4—C5—C6—C7	−178.16 (16)	C12—C13—C14—O6	176.28 (16)
C4—C5—C6—C1	1.1 (3)	O6—C14—C15—C10	−178.98 (17)
C5—C6—C7—O3	−0.8 (2)	C13—C14—C15—C10	1.2 (3)

### Hydrogen-bond geometry (Å, °)

**Table:**

*D*—H···*A*	*D*—H	H···*A*	*D*···*A*	*D*—H···*A*
C5—H5···O3	0.93	2.36	2.710 (3)	102
C8—H8···O1	0.93	2.13	2.798 (2)	127
C9—H9···O3	0.93	2.48	2.797 (2)	100
C11—H11···O3^i^	0.93	2.4	3.306 (2)	164
C17—H17B···O4^ii^	0.96	2.52	3.455 (2)	165
C19—H19C···O4	0.96	2.45	3.013 (2)	117

Symmetry codes: (i) *x*−1/2, −*y*+1/2, −*z*+1; (ii) *x*+2, *y*+1, *z*.

## References

[bb1] Allen, F. H., Kennard, O., Watson, D. G., Brammer, L., Orpen, A. G. & Taylor, R. (1987). *J. Chem. Soc. Perkin Trans. 2*, pp. S1–S19.

[bb2] Brandenberg, K. & Putz, H. (2005). *DIAMOND* Crystal Impact GbR, Postfach 1251, D-53002, Bonn, Germany.

[bb3] Bruker (2008). *APEX2*, *SAINT-Plus*, *X-PREP* and *SADABS* Bruker AXS Inc., Madison, Wisconsin, USA.

[bb4] Chemla, D. S. & Zyss, J. (1987). *Nonlinear Optical Properties of Organic Molecules and Crystals.* Boston: Academic Press.

[bb5] Farrugia, L. J. (1999). *J. Appl. Cryst.* **32**, 837–838.

[bb6] Goto, Y., Hayashi, A., Kimura, Y. & Nakayama, M. (1991). *J. Cryst. Growth*, **108**, 688–698.

[bb7] Kitaoka, Y., Sasaki, T., Nakai, S., Yokotani, A., Goto, Y. & Nakayama, M. (1990). *Appl. Phys. Lett.* **56**, 659.

[bb8] Kraus, G. & Roy, S. (2008). *J. Nat. Prod.* **71**, 1961–1962. 10.1021/np800423j 18855445

[bb9] Marais, J. P. J., Ferreira, D. & Slade, D. (2005). *Phytochem.* **66**, 2145–2176. 10.1016/j.phytochem.2005.03.006 16153413

[bb10] Patil, P. S., Teh, J. B.-J., Fun, H.-K., Razak, I. A. & Dharmaprakash, S. M. (2006). *Acta Cryst.* E**62**, o896–o898.

[bb11] Pietta, P., Gardana, C. & Pietta, A. (2003). *Flavonoids in Health and Disease* edited by C. A. Rice-Evans & L. Packer, 2nd ed. New York: Marcel Dekker, Inc.

[bb12] Rosli, M. M., Patil, P. S., Fun, H.-K., Razak, I. A. & Dharmaprakash, S. M. (2006). *Acta Cryst.* E**62**, o4228–o4230.

[bb13] Sheldrick, G. M. (2008). *Acta Cryst.* A**64**, 112–122. 10.1107/S0108767307043930 18156677

[bb14] Teh, J. B.-J., Patil, P. S., Fun, H.-K., Razak, I. A. & Dharmaprakash, S. M. (2006). *Acta Cryst.* E**62**, o890–o892.

[bb16] Tonder, J. H. van, Muller, T. J. & Bezuidenhoudt, B. C. B. (2010). *Acta Cryst.* E**66**, o1798–o1799. 10.1107/S1600536810022142 PMC3007056 21588008

[bb15] Uchida, T., Kozawa, K., Sakai, T., Aoki, M., Yoguchi, H., Abdureyim, A. & Watanabe, Y. (1998). *Mol. Cryst. Liq. Cryst.* **62**, 135–140.

[bb17] Zhang, G., Kinoshita, T., Sasaki, K., Goto, Y. & Nakayama, M. (1990). *J. Cryst. Growth* **100**, 411–416.

